# How domestication, feralization and experience-dependent plasticity affect brain size variation in *Sus scrofa*

**DOI:** 10.1098/rsos.240951

**Published:** 2024-09-18

**Authors:** T. Cucchi, D. Neaux, L. Féral, F. Goussard, H. Adriensen, F. Elleboudt, G. Sansalone, R. Schafberg

**Affiliations:** ^1^ Archéozoologie, Archéobotanique: Sociétés, Pratiques et Environnements, Muséum National d’Histoire Naturelle CNRS, Paris UMR 7209, France; ^2^ Centre de Recherche en Paléontologie – Paris (CR2P), Muséum National d’Histoire Naturelle, Paris, France; ^3^ PIXANIM, UMR Physiologie de la Reproduction et des Comportements, INRAE, CNRS, Université de Tours, Nouzilly, France; ^4^ Department of Life Sciences, University of Modena and Reggio Emilia,, Modena, Via Campi 213D 41125, Italy; ^5^ Function, Evolution and Anatomy Research Lab, Zoology Division, School of Environmental and Rural Science, University of New England, Armidale, New South Wales, Australia; ^6^ Central Natural Science Collections, Martin-Luther University Halle-Wittenberg, Halle (Saale), Germany

**Keywords:** brain, endocranial volume, domestication, feralization, captivity

## Abstract

Among domestic species, pigs experienced the greatest brain size reduction, but the extent and factors of this reduction remain unclear. Here, we used the brain endocast volume collected from 92 adult skulls of wild, captive, feral and domestic *Sus scrofa* to explore the effects of domestication, feralization and captivity over the brain size variation of this species. We found a constant brain volume increase over 24 months, while body growth slowed down from month 20. We observed an 18% brain size reduction between wild boars and pigs, disagreeing with the 30%–40% reduction previously mentioned. We did not find significant sexual differences in brain volume, refuting the theory of the attenuation of male secondary sexual characteristics through the selection for reduced male aggression. Feralization in Australia led to brain size reduction—probably as an adaptation to food scarcity and drought, refuting the reversal to wild ancestral brain size. Finally, free-born wild boars raised in captivity showed a slight increase in brain size, potentially due to a constant and high-quality food supply as well as new allospecific interactions. These results support the need to further explore the influence of diet, environment and experience on brain size evolution during animal domestication.

## 1. Introduction

Brain size evolution in vertebrates is associated with the increase of cognitive performances in ecological and social domains across species [1,2]. The cognitive benefits of larger brain size are linked to spatial memory and the capacity to navigate in a complex and challenging territory, fostering behavioural flexibility and complex foraging techniques to access difficult-to-extract highly nutritious resources [3–6]. Larger brains are also beneficial to live in larger groups, to be more efficient with predation and to deal with complex cooperation [7]. Yet, the brain is one of the most metabolically expensive tissues in the body across all vertebrates, requiring a constant supply of energy. It also implies a longer maturation time, limiting the total lifetime fertility. Evolving a bigger brain to increase cognitive capacities, therefore, is only adaptive when fitness has overcome these trade-offs [2].

Within animal domestication, a new evolutionary force driven by our species has impacted the brain size evolution of vertebrates [8]. Historically, this evolution is seen as a brain size reduction related to the relaxed selection of cognitive capacities in the human environment [9]. The latter would buffer or suppress altogether the need to forage for food, to protect from seasonal and climatic changes, to find mates, to face competition or to avoid predation. This hypothesis attributing brain size reduction with reduced cognitive demands is due to the fact that the increase in brain size, relative brain size (body size corrected) and neuron numbers are associated with increased cognitive capacities [10]. However, more recently, researchers have considered that no universal pattern should be expected in brain size change during domestication [11], while its effect on brain size should be revised altogether [12] and considered as the result of an interaction of drivers and constraints that need to be further understood [13]. Indeed, the brain is the most energy-expensive tissue in the body [2], selection for metabolic investment in faster reproduction, increased fecundity or somatic production (milk, muscle, eggs) are potential components of brain size reduction across different domestic species [13]. Cognitive factors such as behavioural flexibility and learning in a new niche or metabolic factors such as increased stability and enriched diet can also lead to brain size increase in domestic animals [13]. While experience-dependent plasticity within a lifetime could potentially be an important factor in the brain size evolution of domestic mammals, it remains understudied [13].

According to studies performed on domestic species in the 1970s and 1980s, pigs have undergone the greatest brain size reduction up to 40% [8,14]. To explore the different evolutionary, developmental and experiential factors associated with domestication that could influence brain size variation in the *Sus scrofa* species, we used a unique *in vivo* longitudinal record of wild boar skull growth [15] associated with adult population samples of hunted wild boars or those raised in captivity, Australian feral pigs, and pigs from landraces and industrial breeds. To compare these samples, we measured the endocast volume (ECV) as a brain size proxy [16]. To correct for overall body size differences among the *Sus scrofa* populations sampled, we calculated a relative brain size using the foramen magnum breadth (FMb) measured from the skull as a body size proxy [17]. FMb has been used recently to measure the relative brain size in a review on the effect of domestication across several mammalian taxa, *Sus scrofa* included [14]. The advantage of this proxy is the possibility to obtain this data from fossil and sub-fossil crania, when they are well preserved, to explore the relative brain size change across the long temporal depth of the domestication history.

We first assessed the effects of ontogeny, sex differences and ageing in wild and domestic pigs on ECV variation. Second, the effects of domestication over ECV changes were tested by comparing wild boar populations and pig samples with different selective breeding lines—we hypothesized that breeding selection for faster growth and greater muscle mass production would have induced a brain size reduction compared with hunted wild boars. Third, we evaluated the feralization effect over brain size evolution by comparing hunted wild boars, farmed pigs and feral pigs from Australia. The feral pigs are descended from pigs introduced in the mid-nineteenth century. Recent studies have shown that domestic populations returned to the wild retain their small brain [18]. However, exceptions in the feral pigs of Sardinia [19], Australian dingoes [20] and feral minks [21] suggest that the role of adaptation to a shifting niche varies between species and relative contexts [13]. Here, we hypothesized that 200 years of feralization in an environment the size of Australia with no natural predators could have fostered behavioural flexibility and cognitive demands in feral pig populations that led to an adaptive brain size increase. Finally, we considered the experience/environmental effect of captivity and its plastic response in wild boars relying on an experiment which performed *in vivo* longitudinal computed tomography (CT) scans on 24 male and female wild boars caught in the wild at six months old and raised in captivity until 25 months, when sexual dimorphism is fully expressed (http://anr-domexp.cnrs.fr/). Captivity as a factor of brain size reduction has been observed for many mammals that have lived in captivity for generations [22]. In contrast, urban life has been shown to increase cranial capacities in rodents [23], suggesting important plastic changes in brain size. Here, we hypothesized that the drastic reduction in cognitive demand and territoriality challenge during growth in captivity would have induced a plastic reduction of brain size in free-born wild boar piglets raised in captivity until adulthood.

## 2. Material and methods

### 2.1. Sampling and selection of specimens

We assessed the confounding effects of domestication, feralization and environmental plasticity over the brain size variation in *Sus scrofa* using 92 adult specimens aged between 13 and 70 months of age divided into seven grouped samples (electronic supplementary material, file S1; table 1). The first group consisted of 24 wild boars (*Sus scrofa scrofa*) from Northern France; the second and third groups comprised 22 free-born wild boars (*S. s. scrofa*) captured in Northern France and raised in two different captive environments (12 in a 3000 m^2^ enclosure and 10 in a 100 m^2^ stall; for further detail about the experiment visit http://anr-domexp.cnrs.fr/). The fourth, fifth and sixth groups are pigs (*Sus scrofa domesticus*) from different breeding lines. We have included so-called Landraces from Germany [14] and from the Corsican island [7]. According to Merriam-Webster’s definition landraces are ‘a local variety of a species of plant or animal that has distinctive characteristics arising from development and adaptation over time to conditions of a localized geographic region and that typically displays greater genetic diversity than types subjected to formal breeding practices’. The Corsican Landrace is a small-sized breed adapted to an extensive rearing system based upon the use of local resources like pastureland, chestnuts and acorns [24]. We have also included six Berkshire pigs, a traditional British breed. Finally, the seventh group included 18 feral pigs from the Northern Territory of Australia. The precise origin of these feral populations is still uncertain, but it is accepted that European and Asian domestic pigs were released in Australia by the first European settlers, probably in Sydney in 1788 [25]. In the Northern Territory, pig populations from China were probably introduced during the Gold Rush [25,26]. Pigs were also imported to the Northern Territory from the islands of Timor in 1827 and Kisar in 1838 [25,26]. Some of these domestic pigs were then either released on purpose or escaped and established feral populations [26,27].

**Table 1.** Origin, sample size and sex ratio of the adult *Sus scrofa* dataset. Abbreviations: WB = hunted wild boars, CWB = captive wild boar, DP = domestic pigs and FP = feral pigs. MNHN = Muséum National d’Histoire Naturelle (Paris, France); NMA = National Museum of Australia (Canberra, Australia); ZNS = Central natural science collection (Halle/Saale, Germany). For specimen details and measurements see electronic supplementary material, file S1.

samples	groups	*N*	sex ratio	localization	curation
Hunted Wild boars (*S. s. scrofa*)	WB	24	13 M, 11 F	France	MNHN
Captive wild boars raised in enclosure (*S. s. scrofa*)	CWB	12	5 M, 7 F	France	MNHN
Captive wild boars raised in stall (*S. s. scrofa*)	CWB	10	5 M, 5 F	France	MNHN
European pig Landrace (*S. s. domesticus*)	DP	14	7 M, 7 F	Germany	MNHN/ZNS
Corsican pig Landrace (*S. s. domesticus*)	DP	7	2 M, 5 F	Corsica	MNHN/ZNS
Berkshire pig breed (*S. s. domesticus*)	DP	6	4 M, 2 F	UK	ZNS
Australian feral pigs (*S. scrofa*)	FP	18	12 M, 6 F	Australia	NMA

Our dataset also includes the *in vivo* longitudinal record of the endocranial growth of the 22 captive wild boars described previously, which include the same male and female ratios. The CT scan for the *in vivo* longitudinal observation (LO) was performed at 6, 8, 11, 14, 20 and 25 months of age. The body weight (kg) has been recorded before each LO for all the specimens. Unfortunately, we have not been able to obtain the endocast of all specimens at each LO, which explains why we do not have 22 specimens for each LO (see electronic supplementary material, file S2).

Experiments using captive wild boars adhered to all the ethical agreements (APAFIS#5353-201605111133847).

### 2.2. Data acquisition

CT skull scans were undertaken at several facilities (PIXANIM medical CT for French wild boars and experimental specimens, Halle medical CT for the Halle pigs and the I-MED Radiology Network for the feral pigs from Australia).

The three-dimensional skull meshes were obtained with Avyzo 8.

**Figure 1.**
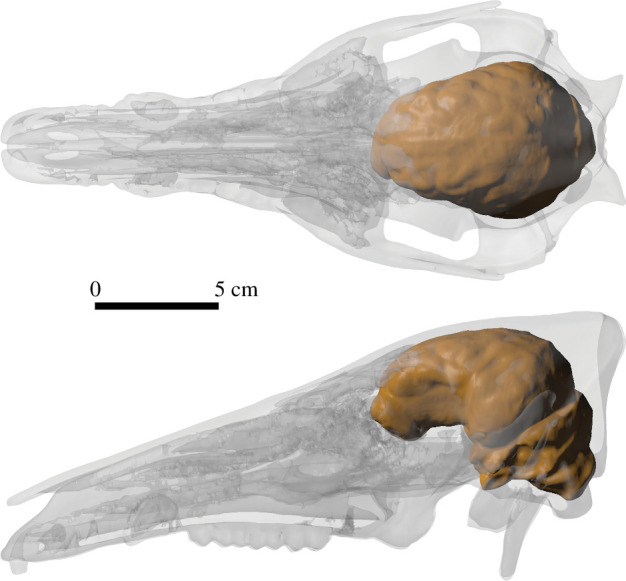
Volume-rendered skull model and the endocast (brown) of a wild boar (*Sus scrofa*) in dorsal (top) and lateral (bottom) views.

Endocast volume in cm^3^ (ECV) were obtained from the three-dimensional skull mesh using the R script from *Endomaker* library (Profico *et al*. [28]) (figure 1).

For each three-dimensional skull mesh, we measured the foramen magnum breadth (FMb) as a body size proxy [17]. To obtain a body size-free brain volume (relative brain size) we used the regression’s residuals of the log-transformed brain volume on log-transformed FMb.

### 2.3. Statistics

Due to the small sample size, we tested the distribution of the logECV using the Shapiro–Wilk’s test in order to choose the appropriate statistical tests. The Shapiro–Wilk’s test performed separately on the adult dataset and on the longitudinal record of growth showed that the logECV distribution did depart from normality for the adult dataset (*W* = 0.96239, *p*‐value = 0.009972) but not for the longitudinal growth record (*W* = 0.98399, *p*‐value = 0.3216); therefore, we used non-parametric tests for statistical analyses on the adults’ brain size dataset and parametric statistics for the growth longitudinal dataset.

The percentage of size differences between each group was calculated using the median rather than the mean values due to the difference in sample sizes.

The variation in the endocast volume (logECV) has been graphically displayed on a violin boxplot (figure 3). To assess the differences in brain size and relative brain size variation among the seven groups of the adult dataset, we performed non-parametric Kruskal–Wallis test and Wilcox pairwise comparisons between group levels with corrections for multiple testing (Benjamini–Hochberg (BH) adjustment method). To compare ECV variation among the growth stages of the longitudinal dataset, we used a pairwise *t*‐test comparison with Bonferroni correction.

To assess the relationship between endocast volume and foramen magnum breadth, we used a linear regression model with the logECV as a dependent variable and FMb as independent variable. To evaluate how much domestication has influenced the variation of the volume and relative volume of the endocast, we used a factorial ANOVA with the logECV as a dependent variable and the three grouping factors (wild boars, European Landrace, English breed/Berkshire) as independent variables.

Statistical analyses were performed with R v. 4.3.2. and R Studio v. 2022 12.2. using the packages ‘dplyr’ v. 2.4.0, ‘ggstaplot’ [29] and ‘ggplot2’ [30].

## 3. Results

### 3.1. Brain size growth in male and female wild boars according to captivity contexts and sexual differences

**Figure 2.**
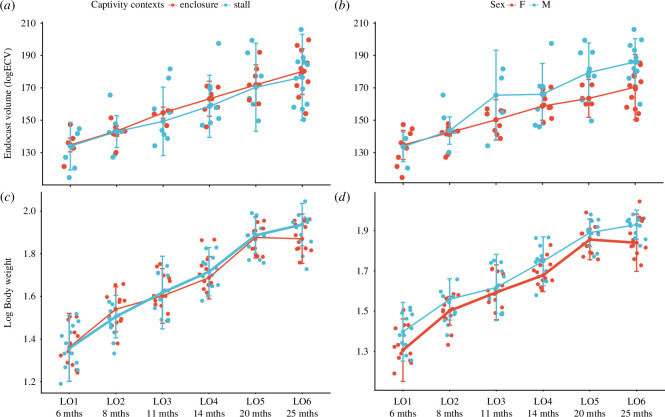
The *in vivo* longitudinal observations (LO) of growth in endocast volume (logECV) and body weight (LogWeight) in wild boars from 6 to 25 months, taking into account (*a,c*) the captivity contexts (enclosure = 3000 m^2^, stall = 100 m^2^) and (*b,d*) the difference between males and females.

The *in vivo* longitudinal record of wild boar brain size growth in a controlled environment (figure 2*a*) shows a 33% increase in the endocranial volume from 6 to 25 months in wild boars without any sign of inflexion during this growth range according to pairwise comparisons (table 2A), while their body mass growth (figure 2*c*) seems to slow down from 20 months (table 2B).

**Table 2.** Pairwise *t*‐test comparison with Bonferroni adjustment method between each longitudinal observation (LO) of (A) brain endocast volume (logECV) and (B) body weight (LogWeight) for the experimental captive wild boars (*Sus scrofa scrofa*). Non-significant *p* values are in bold.

A					
	LO1	LO2	LO3	LO4	LO5
LO2	**0.8665**	—	—	—	—
LO3	0.0024	**0.5757**	—	—	—
LO4	6.30 × 10^−7^	0.0016	**1.0000**	—	—
LO5	4.70 × 10^−11^	2.60 × 10^−7^	0.0026	**0.3236**	—
LO6	3.90 × 10^−15^	9.60 × 10^−11^	1.80 × 10^−5^	0.0096	**1.0000**

B					
	LO1	LO2	LO3	LO4	LO5
LO2	1.90 × 10^−7^	—	—	—	—
LO3	<2 × 10^−16^	0.00055	—	—	—
LO4	<2 × 10^−16^	1.00 × 10^−12^	0.00119	—	—
LO5	<2 × 10^−16^	<2 × 10^−16^	<2 × 10^−16^	2.40 × 10^−8^	—
LO6	<2 × 10^−16^	<2 × 10^−16^	<2 × 10^−16^	1.40 × 10^−10^	**1.0000**

We found no difference in brain growth between the two contexts of mobility constraints (figure 2*a*, enclosure versus stall) while a reduction in body mass is observed between the two contexts from 20 months (figure 2*c*). Sexual difference in brain volume was observed from 11 months (figure 2*b*) with an 8.4% endocast size reduction in females compared with males reaching 25 months, while body mass differences between the two sexes (figure 2*d*) are already in place at 6 months and seem to increase from 20 months.

We found no significant influence of age over brain size variation across adult wild, domestic, feral or captive samples (adjusted *R*^2^ = 0.002783, *F*-statistic = 1.251, *p*‐value = 0.2663). Age variation in our adults’ dataset is therefore not a confounding factor in the following analyses.

### 3.2. Sexual brain size differences in wild boars, domestic and feral pigs

Overall, we found no sexual brain size difference among the wild and domestic adult dataset (Kruskal–Wallis *χ*^2^ = 0.20025, d.f. = 1, *p*‐value = 0.6545), disagreeing with the results described above for the growth dataset on wild boars, even when assessed separately in adult European and Corsican pigs (Kruskal–Wallis *χ*^2^ = 0.55641, d.f. = 1, *p*‐value = 0.4557), Australian feral pigs (Kruskal–Wallis χ^2^ = 0.30682, d.f. = 1, *p*‐value = 0.5796) or in adult wild boars (Kruskal–Wallis *χ*^2^ = 0.30682, d.f. = 1, *p*‐value = 0.5796).

### 3.3. Brain size differences among wild, captive, feral and domestic *Sus scrofa*

**Figure 3.**
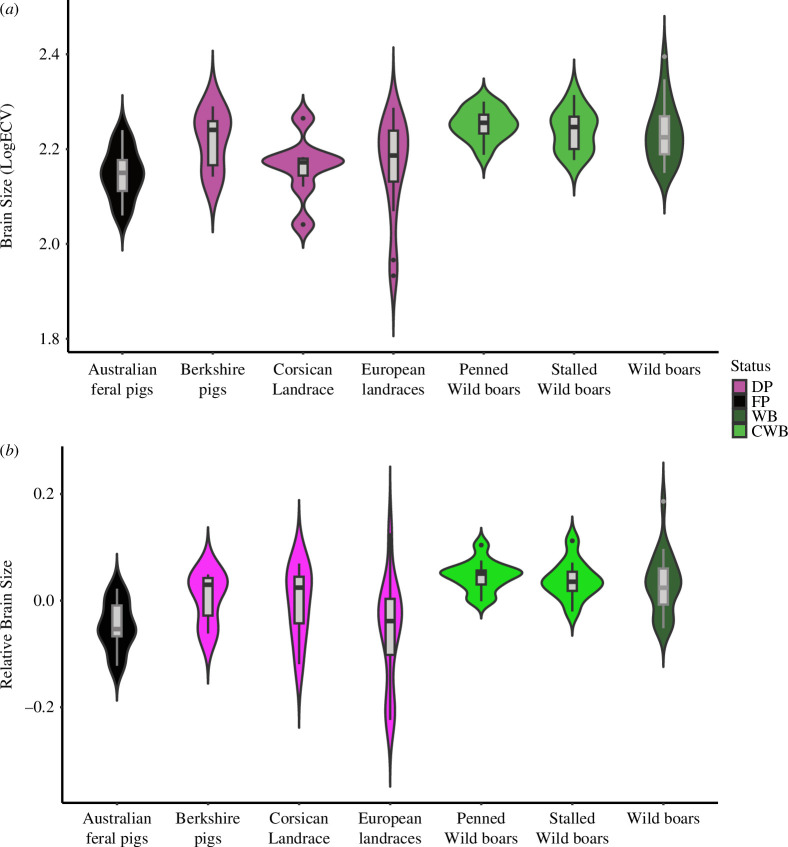
Violin boxplot comparing variation in (*a*) brain volume (logECV), (*b*) brain relative volume (regression’s residuals of log-transformed brain volume on log-transformed FMb) between hunted wild boars (WB), captive wild boar (CWB), domestic pigs (DP) and feral pigs (FP) samples of *Sus scrofa*.

We found significant differences among wild, captive, feral and domestic *Sus scrofa* (figure 3*a*,*b*), in terms of both brain volume (Kruskal–Wallis *χ*^2^ logECV = 30.594, d.f. = 6, *p* < 0.0001) and relative brain volume (Kruskal–Wallis *χ*^2^ = 35, d.f. = 6, *p* < 0.0001). We found a 13% brain volume difference between wild boars (hunted and captive) and all domestic pig breeds. This reduction reached 18% when the small Corsican Landrace and captive wild boars are excluded in order to only compare continental hunted wild boars versus non-insular pigs. Despite these overall differences, we found no significant brain or relative brain volume differences between wild boars and domestic breeds when using the Wilcox pairwise comparisons (table 3). Significant brain size and relative brain size reduction are only observed between wild boars and the feral pigs from Australia and the small Corsican Landrace (table 3). We observed that Berkshire pigs display an endocast volume that is 12% larger than Landrace pigs, yet this difference is not significant (table 3).

**Table 3.** Wilcox pairwise comparisons with BH adjustment method of (A) the brain endocast volume (logECV) and (B) the relative brain size (residuals) between seven S*us scrofa* groups. Significant differences have been written in bold.

A.	brain endocast volume (logECV)					
	Australian feral pigs	Berkshire pigs	Corsican landrace	European landraces	Penned wild boars	Stalled wild boars
Berkshire pigs	0.08166	—	—	—	—	—
Corsican landrace	0.60163	0.44231	—	—	—	—
European landraces	0.37492	0.44231	0.74262	—	—	—
Penned wild boars	**0.00058**	0.45486	**0.01375**	**0.03045**	—	—
Stalled wild boars	**0.00121**	0.70225	**0.03045**	0.08166	0.69612	—
Wild boars	**0.00077**	0.74262	0.07438	0.14568	0.386	0.69612

B.	Relative **b**rain **s**ize (residuals)					
	Australian feral pigs	Berkshire pigs	Corsican landrace	European landraces	Penned wild boars	Stalled wild boars
Berkshire pigs	0.06731	—	—	—	—	—
Corsican landrace	0.14485	1	—	—	—	—
European landraces	0.81776	0.22961	0.30058	—	—	
Penned wild boars	**1.50 × 10^−5^**	0.19382	0.30058	**0.00167**	—	
Stalled wild boars	**0.00021**	0.61895	0.37079	**0.00627**	0.66612	
Wild boars	**5.80 × 10^−5^**	0.57954	0.61895	**0.00804**	0.37248	0.66612

**Figure 4.**
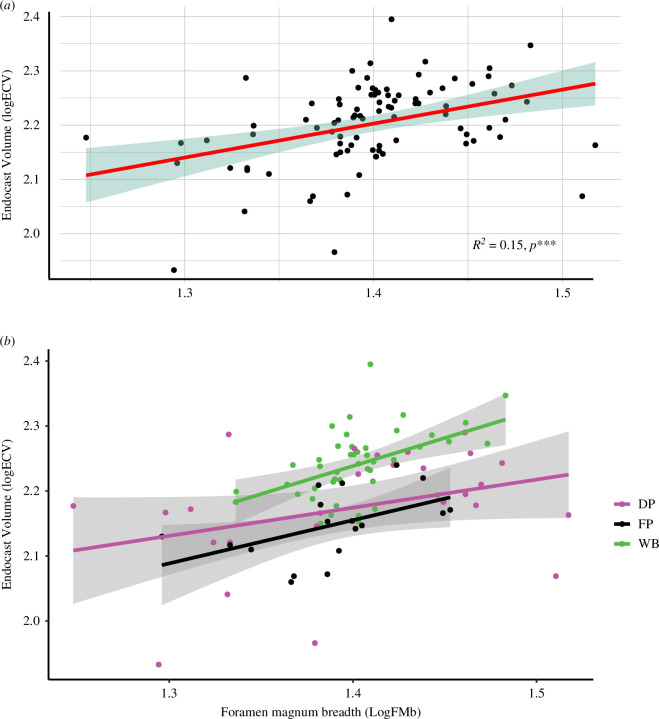
Relative brain size in *Sus scrofa* using the regression of endocast volume (logECV) versus the foramen magnum breadth (logFMb) as body mass proxy: (*a*) across all the *Sus scrofa* specimens with regression slope and its interval of confidence; (*b*) separating wild (WB), feral (FP) and domestic (DP) *Sus scrofa* with respective regression slopes and confidence interval.

We also found significant (multiple *R*^2^ = 0.15, *F*-statistic = 15.58, *p* < 0.001) but weak linear relationships between the endocast volume and the cranium size for all the wild, captive, feral and domestic samples (figure 4*a*). Yet wild boars (hunted or captive) and feral pigs showed a greater brain to body size allometry than domestic pig samples (figure 4*b*).

ANOVA found a small but significant influence of selective regimen over the brain volume (adjusted *R*^2^ = 0.1259, *F*-statistic = 3.4, *p* = 0.02524) and the relative brain volume (adjusted *R*^2^ = 0.168, *F*-statistic = 4.297, *p* = 0.00937) when using wild boars (captive excluded), European domestic Landraces, Corsican Landraces and Berkshire pigs as grouping factors for the linear models.

### 3.4. Feralization and change in brain size

Feral pigs from Australia showed a significant difference in their brain volume (14%) and relative brain volume compared with wild boar samples but no significant reduction of brain volume or relative brain volume compared with pig samples from Landraces, or Berkshire breed (figure 3*a*,*b* and table 3).

### 3.5. Captivity experience during growth and brain size change in wild boar

The brain volume variation and the relative brain volume variation of wild boars that were raised in captivity is not significantly different from wild boars hunted in their natural habitat (figure 3*a*,*b*). However, we observe that the ECV median of captive specimens is 6% larger than the wild boar ECV.

## 4. Discussion

Differences in brain size between wild and domestic species have been documented in evolutionary science for more than a century, but the relevance of these changes has recently been challenged [31] and many confounding factors remain to be disentangled—such as ageing changes and sexual differences and neuroplastic response to environmental changes. In this study, we explored the effects of these factors on the brain size variation of *Sus scrofa*, a model species considered to have undergone the greatest brain reduction among domesticated mammals [14]. The endocast’s volume was extracted automatically from skull CT scans as a proxy of the brain size and we implemented a body size correction using the foramen magnum breadth.

### 4.1. Effects of age and sexual differences on brain size

Studies on pig brain development do not go beyond six months [32,33]; in this study, however, we provide the first long-lasting *in vivo* longitudinal record of brain growth in a large mammal until adulthood. Thanks to this record, we found that *Sus scrofa* brain volume increases steadily until 25 months with a sexual difference in brain size already in place from the 11th month. Brain growth and sexual differentiation in brain volume does not seem to be fully aligned with the body mass growth of the captive specimens which slows down from 20 months and shows sexual differences from 6 months. In their natural habitat, wild boars reach 75% and more of their body weight by 24 months and could continue growing in males over 36 months, while sexual difference in body size starts by the 20th month [34]. Unfortunately, our *in vivo* longitudinal record could not be carried out until 36 months so we cannot exclude that the brain growth could slow after 25 months. However, these results suggest that wild boar brain growth is steady between 6 and 25 months, despite sexual maturity and the inflection of the body growth curve from 20 months.

We also found no ageing influence over brain size variation in a cohort of wild and domestic adults, suggesting that age variability should not be considered as a potential bias when comparing the brain size variation of adult *Sus scrofa* across wild and domestic populations.

We found a 6% brain volume difference between 25-month-old male and female wild boars that have grown in captivity for the experiment, but no significant sexual difference was detected in the adult dataset of wild boars, domestic pigs and feral pigs. Sexual differences in the brain volume of the captive wild boars is probably related to the body mass difference (19%) between males and females at 25 months old (see [35] for body mass details). Yet, we did not find significant sexual change in brain volume in wild and domestic *Sus scrofa*, refuting the theory of the attenuation of male secondary sexual characteristics through the selection for reduced male aggression as a driver for brain size reduction in females [13]. The greater sexual expression observed for the experimental dataset could be explained by a reduced access to the food supply for the females, which could be due to greater male aggressiveness; unfortunately, we did not record this information during the experiment. More species-specific empirical studies are therefore required to further address this model.

### 4.2. Selective regime and brain size changes

To assess the effect of domestication on brain size, we compared brain volume and body size corrected brain volume between European wild boars, European Landraces (German and Corsican) and the Berkshire breed. We found a significant effect of the selective regime on *Sus scrofa* brain size variation with an 18% reduction trend between European wild boars and European domestic pigs.

Our study cannot answer the important question of whether the smaller brain of pigs compared with wild boars is inherited from the early process of reproductive control during domestication or the result of the recent breeding selection. The Berkshire breed selected for muscle mass production showed a trend towards a 12% increase in brain volume compared with Landraces, contradicting our assumption that selective breeding, leading to the increase of metabolic investment in muscle tissue (meat), tends to reduce the energy investment for brain development [13]. The slight increase in brain size of Berkshire pigs compared with traditional Landraces would suggest that this smaller brain size compared with wild boars could be inherited from the first phase of reproductive control during the early process of pig domestication. Only a bioarchaeological approach could provide the right comparative material to answer this crucial question.

The 18% brain size reduction observed agrees with previous results by Röhrs & Ebinger [36] and Balcarcel *et al*. [14], despite a different brain size approximation and a different statistical approach for brain size differences between wild and domestic animals. This further undermines the brain size reduction between 30% and 40% previously considered for domestic pigs [8]. Yet, the reduction we observed using the volume of the brain endocast is not statistically significant, suggesting that further studies are required with larger samples of wild boar populations across Eurasia and a greater diversity of domestic pigs including Chinese breeds.

### 4.3. Feralization effect on *Sus scrofa* brain size

Despite their great evolutionary interest [37], studies on the effect of feralization on animal phenotypes and especially brain anatomy are still scarce [18]. The seminal work on the feral pig populations from the Galapagos Islands [38], showed that Galapagos pigs had a 30% smaller relative brain size compared with wild boars. This has also been observed for dogs, rodents, birds, fish [39] and even for ungulates that have been feral for thousands of years, such as the Mediterranean sheep [40]. However, some exceptions have recently been reported, such as the dingo [20] or feral populations of mink [21], which both display a reverse movement towards a larger brain. Feral pigs of the island of Sardinia also display a larger brain than wild boar and domestic pigs from the island [19], suggesting that brain size could be one of the reversible traits of domestication through feralization, depending on the species and environmental context.

In this study, we found that the descendants of the first Australian feral pigs did not regain the brain size of continental wild boars. This contradicts our assumption that 200 years of feralization in a new challenging environment [41] the size of Australia, requiring more behavioural flexibility, would require an adaptive increase in the brain size [3], inducing a reverse towards wild boar brain size variation. On the contrary, Australian feral pigs display a brain volume in the range of the small-bodied insular Corsican pig Landrace, smaller than nineteenth-century historical pig Landraces (contemporaneous with the introduction of pigs to Australia). Accordingly, the brain size of Australian feral pigs neither reverted towards a ‘wild’ brain as we expected nor remained unreversed from their ancestral domestic traits; rather it has further reduced. A skull size reduction has already been observed from the same Australian feral specimens and comparative pigs [42] and fits the body description of Australian feral pigs as smaller and leaner than pigs [43]. This brain reduction in Australian feral pigs could have been adaptive due to the reduction of the energy cost of a small brain in an environment minimizing anti-predator behaviours [44,45]. Yet, it has been reported that dingoes and feral dogs prey on feral pigs [46], which could undermine this assumption. On the other hand, the high mortality of Australian feral pigs is often associated with starvation and drought [47,48], suggesting that the resource scarcity here is probably the strongest selective pressure that the founding populations of feral pigs had to adapt to, and that body and brain size reduction were the most likely adaptive responses.

### 4.4. Lifetime captivity experience effect on wild boar brain size

The few studies on the effect of captivity on mammal brains considers that captivity-induced brain reduction, due to the degradation of the neural network for functions no longer required in captivity, favour a reduction of the brain’s energy cost [49]. Studies from captive populations living in zoos or reserves for several generations found brain size reduction in lions and tigers [50]. It has been shown, however, that enriched environment for captive animals [51] could lead to larger brains, as shown in laboratory rodents [52,53], where the complexity of the captive environment is a key component of the brain size variation in captivity [22]. More recent studies have provided evidence for the role of developmental neuroplasticity in brain morphology [54,55].

In this study, we have provided the first experimental data for the effect of mobility reduction in developmental neuroplasticity in wild boar. We found no brain size or relative brain size reduction in captive wild boars, suggesting that territoriality reduction, a lack of social interaction and a reduction in cognitive demand for resource access with captivity did not induce a plastic brain size reduction in wild boars. Instead, we observed an increase trend of the brain and relative brain size in captive wild boars, suggesting that the plastic response to captivity was actually directed towards an increase in brain size, contradicting our assumption that the loss of cognitive demand in captivity would induce a brain size reduction. One potential explanation for this plastic increase would be the stability and quality (rich in protein) of the food supply in our experimental setting, as suggested by the brain size increase of captive wolves due to high-quality nutrition [56]. Another explanation is that the lack of behavioural flexibility in captivity was balanced by the need to learn in a new niche where the experimental specimens had an enriched environment to prevent stress and were regularly in contact with human carers. This interpretation would be in line with the experiments on laboratory rats where specimens raised in enriched environments had larger brains compared with standard laboratory rats [52] and a greater number of neurons in the hippocampus of house mice raised in stimulating conditions [53]. Therefore, we propose that the stable diet-rich captive environment associated with a new environment and new allospecific interactions could have had neurodevelopmental influence on certain brain region size via synapse formation and cell and synapse pruning [13,57].

## 5. Conclusion

*Sus scrofa*, is considered to be the species that have undergone the greatest brain size reduction among domesticated species. Our study of brain endocast volume supports a brain size reduction up to 18% but rejects previous claims of brain reduction up to 40%. The increase in artificial selection for muscle mass production in this species does not seem to have induced the expected brain size reduction and suggests that reproductive control during the early process of domestication should be further investigated as drivers. Archaeological skulls of *Sus scrofa* from both the Near Eastern and the Chinese domestication centres would undoubtedly help to understand the respective roles of early reproductive control and later breeding selection in the brain evolution of this species. In Australian feral pig populations, the return to natural selection in a challenging environment has not induced the expected return to the wild-sized brain. We propose that an adaptive response to food scarcity for generations has further reduced the brain size inherited from their domestic ancestors. Finally, wild boars raised in captivity until adulthood display a slight increase in brain size, which contradicts the expectation of a cognitively less demanding captive environment. We propose that the constant supply of high-quality food and an enriched environment could have counterweighted the effect of a drastic reduction on cognitive demands by influencing new neurodevelopmental processes. These results suggest that environment and experience are drivers that need further investigations to better understand brain size evolution during domestication.

## Data Availability

The data is available at [58]. Supplementary material is available online [59].
